# S76. A BEHAVIOURAL ECONOMIC ANALYSIS OF EFFORT-RELATED CHOICE IN SCHIZOPHRENIA

**DOI:** 10.1093/schbul/sby018.863

**Published:** 2018-04-01

**Authors:** Gagan Fervaha, Ofer Agid, George Foussias, Hiroyoshi Takeuchi, Konstantine Zakzanis, Ariel Graff-Guerrero, Gary Remington

**Affiliations:** 1 Queen’s University Faculty of Medicine; 2 Centre for Addiction and Mental Health (CAMH), University of Toronto; 3 University of Toronto

## Abstract

**Background:**

Motivational deficits are prevalent feature of schizophrenia, which have been tightly linked to real-world outcomes. Abnormalities in effort cost computations have been proposed as a candidate mechanism underlying these deficits. In the present study, we sought to employ behavioural economic analyses to further understand cost-benefit decision making abnormalities in schizophrenia.

**Methods:**

58 young adults with schizophrenia and 58 matched controls participated in this study. Participants completed an effort-based decision-making task in which they made decisions to expend physical effort in exchange for monetary rewards. From participants choice behaviour, we computed indifference values (i.e. the reward value at which participants would be indifferent to expending effort vs not) for each individual participant. Other computational parameters were also computed such as choice consistency and subjective reward valuation.

**Results:**

Patents and controls did not differ in their subjective valuation of reward. On the decision-making task, patients made more inconsistent choices relative to controls. In both univariate and multivariate analyses controlling for potential confounders, patients had higher indifference values meaning that patients required more money in the exchange of their effort. Among patients, higher indifference values were associated with more severe clinical motivational deficits.

**Discussion:**

Patients had multiple abnormalities related to their decisions to expend effort for reward. Choices were more chaotic and reward value was discounted by effort at a steeper rate in patients. These results point toward an abnormality in the computation of effort costs or in the integration of these costs with value signals.

